# ER Stress Regulates HIV Tat and Alcohol‐Induced NLRP6 Activation in Astrocytes

**DOI:** 10.1002/cns.71184

**Published:** 2026-09-28

**Authors:** Seema Singh, Elias Horanieh, Shilpa Buch, Palsamy Periyasamy

**Affiliations:** ^1^ Department of Pharmacology and Experimental Neuroscience University of Nebraska Medical Center Omaha Nebraska USA

**Keywords:** astrocytes, ER stress, ethanol, HIV tat, NLRP6 signaling

## Abstract

**Introduction:**

Chronic alcohol consumption is common among people living with HIV (PLWH) and exacerbates HIV‐associated neurocognitive disorders (HAND). HIV‐1 transactivator of transcription (Tat) and ethanol synergistically promote neuroinflammation, but the underlying molecular mechanisms remain unclear. This study aimed to determine whether endoplasmic reticulum (ER) stress regulates astrocytic NLRP6 inflammasome activation in response to HIV Tat and ethanol coexposure.

**Methods:**

Mouse primary astrocytes were exposed to HIV Tat (50 ng/mL) and ethanol (50 mM), individually or in combination. Astrocyte activation, NLRP6 inflammasome signaling, proinflammatory cytokine production, and ER stress responses were assessed using western blotting, immunocytochemistry, qPCR, and ELISA. ER stress was inhibited using 4‐phenylbutyric acid (4‐PBA), and NLRP6 was silenced using siRNA to define pathway hierarchy.

**Results:**

Combined HIV Tat and ethanol exposure markedly increased astrocytic activation, NLRP6 expression, caspase‐1 cleavage, and IL‐1β and IL‐18 production compared with individual treatments. Coexposure also significantly induced ER stress markers BiP, ATF6, and phosphorylated eIF2α. Pharmacological inhibition of ER stress suppressed NLRP6 inflammasome activation and astrocytic reactivity, whereas NLRP6 knockdown attenuated inflammatory signaling without affecting ER stress markers, indicating ER stress acts upstream of NLRP6.

**Conclusion:**

These findings identify an ER stress–NLRP6 inflammasome axis as a potential mechanism underlying HIV Tat and ethanol‐induced astrocytic neuroinflammation, providing mechanistic insight into alcohol‐associated neuroinflammatory processes relevant to HAND.

## Graphical abstract

HIV Tat and ethanol synergistically induce ER stress in astrocytes, activating an ER stress‐NLRP6 inflammasome axis that drives caspase‐1 activation and IL‐1β/IL‐18 release. Targeting ER stress or NLRP6 effectively suppresses astrocytic neuroinflammation, identifying a novel therapeutic pathway for alcohol‐exacerbated NeuroHIV.

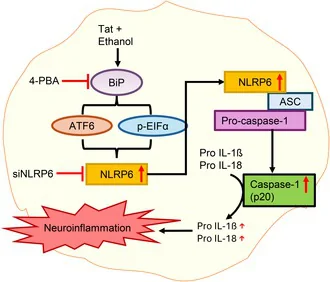

## 1 Introduction

Human immunodeficiency virus (HIV) infection and alcohol use disorder (AUD) frequently co‐occur, posing a major global and national public health burden. According to the World Health Organization, alcohol consumption accelerates HIV disease progression by impairing immune competence and overall health [1]. Epidemiological studies in the United States further reveal that people living with HIV (PLWH) are more likely to engage in hazardous alcohol use, which, in turn, exacerbates immune suppression, enhances viral replication, and increases the risk of neurological and hepatic complications [2, 3]. Chronic alcohol exposure also worsens neurocognitive outcomes, leading to a higher incidence of HIV‐associated neurocognitive disorders (collectively termed NeuroHIV) and other neuropsychiatric manifestations [4]. Thus, addressing mechanistic underpinnings of alcohol misuse in PLWH is crucial for mitigating comorbidities and preserving long‐term neurological health.

Astrocytes, the most abundant glial cell type in the central nervous system (CNS), are essential for maintaining neuronal homeostasis, synaptic function, and neuroimmune balance [5, 6, 7, 8]. Under pathological conditions such as HIV infection or alcohol exposure, astrocytes undergo reactive transformation characterized by upregulation of glial fibrillary acidic protein (GFAP) and secretion of proinflammatory mediators [9, 10, 11, 12, 13]. This astrocytic reactivity disrupts the neuronal microenvironment, promoting chronic neuroinflammation and neurodegeneration, a core feature of NeuroHIV [14, 15, 16, 17]. Astrocyte activation under such stressors is thought to involve innate immune sensors such as inflammasomes, which orchestrate the maturation and release of interleukin‐1β (IL‐1β) and interleukin‐18 (IL‐18), thereby amplifying neuroinflammatory signaling [12, 18, 19].

Among the inflammasome family members, NLRP3 inflammasome has been extensively characterized in glial cells [20, 21, 22], whereas the role of NLRP6 remains largely unexplored in the CNS. NLRP6, traditionally recognized for its contribution to intestinal immune homeostasis and mucosal defense [23, 24], has recently been implicated in sterile inflammation and metabolic regulation, suggesting that it may also participate in neuroimmune signaling [25, 26]. Our previous findings demonstrated that alcohol exposure activates NLRP6 in astrocytes, unveiling a previously unrecognized molecular mechanism by which alcohol potentiates neuroinflammation [12]. The HIV transactivator of transcription (Tat), a neurotoxic viral protein released from infected cells, disrupts mitochondrial integrity [27, 28, 29], and calcium homeostasis [30], resulting in intracellular stress [31]. The co‐occurrence of HIV Tat and alcohol exposure may therefore impose dual stress on astrocytes, resulting in synergistic activation of inflammatory cascades.

Despite mounting evidence implicating inflammasome activation in glial pathology, the upstream regulatory mechanisms connecting cellular stress to NLRP6 signaling in astrocytes remain unknown. Endoplasmic reticulum (ER) stress has emerged as a central mechanism linking environmental and metabolic insults to glial dysfunction [32]. Both HIV Tat and alcohol have been shown to induce the unfolded protein response (UPR) in neural and glial cells, leading to calcium dysregulation, reactive oxygen species generation, and mitochondrial and lysosomal dysfunction [27, 28, 29, 30, 33, 34]. Persistent activation of ER stress pathways, such as PERK, IRE1α, and ATF6, can trigger inflammatory cascades and amplify the release of proinflammatory cytokines [35, 36, 37, 38]. However, whether ER stress serves as an upstream regulator of NLRP6 inflammasome activation in astrocytes exposed to HIV Tat and alcohol remains to be elucidated.

In the present study, we demonstrate that ER stress functions as a key upstream modulator of NLRP6 inflammasome activation in astrocytes. We hypothesize that HIV Tat and alcohol synergistically induce ER stress, which, in turn, triggers NLRP6 activation, astrocytic reactivity, and the release of proinflammatory cytokines. To our knowledge, this is the first study to delineate an ER stress‐NLRP6 inflammasome signaling axis in astrocytes during HIV and alcohol coexposure. Elucidating this pathway provides novel mechanistic insights into the neuroimmune pathology of NeuroHIV in individuals with co‐occurring AUD and identifies ER stress modulation as a promising therapeutic strategy to alleviate HIV Tat and alcohol‐induced neuroinflammation.

## 2 Materials and Methods

### 2.1 Chemicals and Reagents

Endotoxin‐free recombinant HIV‐1 Tat‐101 protein (Cat. No. 1032‐10) was purchased from ImmunoDiagnostics Inc. (Woburn, MA, USA). Ethanol (Cat. No. 2701) was obtained from Decon Labs Inc. (King of Prussia, PA, USA). The Pierce BCA Protein Assay Kit (Cat. No. 23225), protease inhibitor cocktail (Cat. No. 78439), phosphatase inhibitor cocktail (Cat. No. 78427), Opti‐MEM (Cat. No. 31985070), penicillin–streptomycin (Cat. No. 15140122), Verso cDNA Synthesis Kit (Cat. No. AB‐1453/B), and Lipofectamine RNAiMAX (Cat. No. 13778150) were obtained from Thermo Fisher Scientific (Waltham, MA, USA). Silencer negative control siRNA (Cat. No. AM4635) and NLRP6 siRNA (Cat. No. s46905) were also purchased from Thermo Fisher Scientific. Triton X‐100 (Cat. No. BP151‐500) and ProLong Gold Antifade Mountant with DAPI (Cat. No. P36935) were from the same supplier. Non‐fat dry milk (Cat. No. M17200‐1000.0) was purchased from Research Products International (Mount Prospect, IL, USA). Dulbecco's modified Eagle medium (DMEM) containing l‐glutamine, 4.5 g/L glucose, and sodium pyruvate (Cat. No. MT10013CV) and heat‐inactivated fetal bovine serum (FBS; Cat. No. 35‐016‐CV) were obtained from Corning Inc. (Corning, NY, USA). OPI Media Supplement (Cat. No. O5003‐1VL) and L‐leucine methyl ester hydrochloride (Cat. No. L1002‐25G) were purchased from Sigma‐Aldrich (St. Louis, MO, USA). Polyvinylidene difluoride (PVDF) membranes (Cat. No. IPVH00010), anti‐NLRP6 (Cat. No. ABF29), anti‐IL‐18 (Cat. No. 06–1115), and anti‐GFAP (Cat. No. G3893) antibodies were from MilliporeSigma (Burlington, MA, USA). Anti‐caspase‐1 (Cat. No. sc‐398715) and anti‐β‐actin (Cat. No. sc‐47778) were obtained from Santa Cruz Biotechnology (Dallas, TX, USA). Anti‐IL‐1β antibody (Cat. No. ab9722) was from Abcam (Boston, MA, USA). Peroxidase AffiniPure goat anti‐rabbit IgG (Cat. No. 115‐035‐003) and goat anti‐mouse IgG (Cat. No. 111‐035‐003) were from Jackson Immuno Research Laboratories (West Grove, PA, USA). Normal goat serum (Cat. No. S‐1000) was purchased from Vector Laboratories (Newark, CA, USA). Alexa Fluor goat anti‐rabbit 488 (Cat. No. A11008) and goat anti‐mouse 594 (Cat. No. A11005) secondary antibodies were obtained from Invitrogen (Thermo Fisher Scientific, Waltham, MA, USA).

### 2.2 Isolation and Culture of Primary Mouse Astrocytes

Mouse primary astrocytes (MPAs) were isolated from Postnatal Days 0–3 C57BL/6 pups. Following euthanasia by isoflurane, brains were removed, and cortices dissected in pre‐chilled 1× HBSS under sterile conditions to eliminate meninges and blood vessels. Tissues were minced into fine fragments and digested with 5 mL of trypsin–EDTA for 10 min at 37°C with gentle agitation every 2 min. Digestion was halted using 10 mL DMEM supplemented with 10% FBS, and tissues were triturated with glass Pasteur pipettes to obtain single‐cell suspensions. The cell mixture was filtered through a 40 μm strainer and centrifuged at 1000*g* for 5 min at room temperature. Pellets were resuspended in complete astrocyte growth medium containing DMEM, 10% FBS, 1% OPI supplement, 1% l‐leucine methyl ester, and 1% penicillin–streptomycin. Cultures were maintained at 37°C in 5% CO_2_, and medium was replaced every 3–4 days. After 7–10 days, confluent astrocytes were detached using trypsin–EDTA and replated for experiments.

### 2.3 Human A172 Astrocytoma Culture

Human astrocytoma cell line A172 (ATCC CRL‐1620) was obtained from the American Type Culture Collection and maintained according to standard culture conditions. Cells were grown in DMEM supplemented with 10% heat‐inactivated fetal bovine serum and 1% penicillin‐streptomycin. Cultures were maintained at 37°C in a humidified incubator with 5% CO_2_. Cells within Passages 5–25 were used for experiments, and cultures were periodically refreshed from frozen stocks to minimize variability associated with extended passaging.

### 2.4 Western Blotting

At the end of treatments, MPAs and human A172 astrocytes were rinsed with 1× PBS and lysed in RIPA buffer supplemented with protease and phosphatase inhibitors. Lysates were sonicated and centrifuged at 12,000*g* for 10 min at 4°C. Protein concentration was determined using the Pierce BCA Protein Assay Kit, and samples were diluted in 4× Laemmli buffer to a final concentration of 1 μg/μL. Equal amounts of protein were resolved by sodium dodecyl sulfate‐polyacrylamide gel electrophoresis (SDS‐PAGE) and transferred to PVDF membranes. Membranes were blocked with 5% nonfat milk for 1 h at room temperature and incubated overnight at 4°C with primary antibodies diluted in 1× TTBS containing 5% milk. After washing three times in 1× TTBS, membranes were incubated with the appropriate HRP‐conjugated secondary antibodies for 1 h at room temperature. Bands were visualized using the SuperSignal chemiluminescent substrate and imaged with a FluorChem M system (Cell Biosciences, Santa Clara, CA, USA). β‐actin served as the loading control. Band intensities were quantified using NIH ImageJ software [39].

### 2.5 Quantitative Real‐Time PCR (RT‐qPCR)

Total RNA was extracted from MPAs using the Quick‐RNA MiniPrep Plus Kit (Cat No. R1058; Zymo Research, Orange, CA, USA) according to the manufacturer's instructions. RNA purity and concentration were determined using a NanoDrop 8000 spectrophotometer. cDNA was synthesized from 1 μg of total RNA using the Verso cDNA Synthesis Kit. qPCR was performed with SYBR Green chemistry on an Applied Biosystems QuantStudio 3 system (Applied Biosystems, Grand Island, NY, USA). The following mouse primer pairs were used: *Il1β* (FW: CACTACAGGCTCCGAGATGA, RE: TCTGTCCATTGAGGTGGAGA), *Il18* (FW: TAGTCCTTCCTACCCCAATTTCC, RE: TTGGTCCTTAGCCACTCCTTC), and *Gapdh* (FW: GGTGAAGGTCGGTGTGAAGG, RE: CTCGCTCCTGGAGATGGTG). Each reaction was performed in triplicate, and relative gene expression was calculated using the 2^−ΔΔ*CT*^ method, normalized to *Gapdh*.

### 2.6 siRNA Transfection

For gene‐silencing experiments, MPAs were seeded in six‐well plates at 0.5 × 10^6^ cells per well. After 24 h, cells were transfected with 50 pmole of either *Nlrp6* siRNA or scrambled control siRNA using Lipofectamine RNAiMAX (Cat No. 13778150; ThermoFisher Scientific Inc., Pittsburgh, PA, USA) in Opti‐MEM medium according to the manufacturer's protocol. After 12 h, the transfection medium was replaced with complete growth medium, and cells were incubated for 24 h before subsequent treatments with HIV Tat and/or ethanol for an additional 24 h. Transfection efficiency and target knockdown were confirmed by western blotting.

### 2.7 Enzyme‐Linked Immunosorbent Assay (ELISA)

MPAs were seeded in six‐well plates at 0.5 × 10^6^ cells/well, starved overnight in serum‐free medium, and then exposed to HIV Tat (50 ng/mL) and/or ethanol (50 mM) for 24 h. Supernatants were collected and centrifuged at 1000*g* for 10 min at 4°C to remove debris. Clarified supernatants were concentrated using Amicon Ultra‐15 centrifugal filters (Cat. No. UFC901024; MilliporeSigma, Burlington, MA, USA) to ~500 μL and analyzed for IL‐1β and IL‐18 levels using ELISA kits (mouse IL‐1β, Cat. No. ab197742; mouse IL‐18, Cat. No. ab216165; Abcam, Boston, MA, USA) according to the manufacturer's protocol.

### 2.8 Immunocytochemistry

After treatments, MPAs grown on coverslips were washed with PBS and fixed in 4% paraformaldehyde for 20 min at room temperature. Cells were permeabilized with 0.1% Triton X‐100 for 15 min and blocked with 10% normal goat serum for 1 h. Primary antibodies against NLRP6 (1:250) and GFAP (1:500) were applied overnight at 4°C in 10% goat serum. After washing, cells were incubated with Alexa Fluor 488 (goat anti‐mouse) and Alexa Fluor 594 (goat anti‐rabbit) secondary antibodies for 2 h at room temperature. Nuclei were counterstained with DAPI, and coverslips were mounted using ProLong Gold Antifade Mountant. Fluorescent images were acquired using a Zeiss Axio Observer Z1 inverted microscope (Carl Zeiss, Thornwood, NY, USA) and analyzed with AxioVs 40 Version 4.8.0.0 software. Relative fluorescence intensity was quantified using ImageJ software [39].

### 2.9 Statistical Analysis

All experiments were performed in six replicates unless otherwise stated. Data are presented as mean ± SEM. Statistical analyses were conducted using GraphPad Prism version 10 (GraphPad Software, San Diego, CA, USA). One‐way ANOVA followed by Sidak's  multiple comparisons test was used to compare groups. A *p*‐value < 0.05 was considered statistically significant.

## 3 Results

### 3.1 Ethanol Potentiates HIV Tat‐Induced NLRP6 Inflammasome Activation in Astrocytes

To confirm the purity of primary astrocyte cultures, immunofluorescence staining was performed for astrocytic (GFAP), microglial (IBA1), and neuronal (MAP2) markers. Over 97% of DAPI‐positive nuclei colocalized with GFAP, whereas IBA1 and MAP2 signals were undetectable, confirming a highly enriched astrocyte population (Figure S1). Based on our previous studies demonstrating dose‐dependent activation of CNS cells by HIV Tat [40, 41, 42, 43] and astrocytes by ethanol [12], we selected concentrations that consistently induce robust astrocytic activation. Specifically, 50 ng/mL HIV Tat and 50 mM ethanol were used to evaluate their combined effects on NLRP6 inflammasome signaling. Thus, in this study, to investigate whether ethanol enhances HIV Tat‐induced NLRP6 inflammasome activation, MPAs were exposed to HIV Tat (50 ng/mL) and ethanol (50 mM) for 24 h, individually or in combination. Western blot analysis revealed that cotreatment of cells with HIV Tat and ethanol significantly increased the expression of GFAP, a hallmark of astrocytic activation, compared with either treatment alone (Figure 1A). Similarly, NLRP6 protein expression was markedly upregulated following combined exposure (Figure 1B). Immunofluorescence analysis also confirmed these findings, showing robust NLRP6 expression and colocalization with GFAP in HIV Tat and ethanol‐exposed MPAs relative to single exposures (Figure 1C–E). To further evaluate inflammasome activation, we examined downstream signaling mediators, including cleaved caspase‐1 and proinflammatory cytokines. Coexposure to HIV Tat and ethanol significantly increased cleaved caspase‐1 (p20) levels than that of individual exposure (Figure 2A). Quantitative PCR analysis also revealed substantial upregulation of *Il1β* and *Il18* mRNA expression (Figure 2B,C), which was corroborated by elevated protein levels detected via western blotting (Figure 2D–E) and ELISA (Figure 2F–G). These findings were further validated in the human A172 astrocytoma cell line exposed to HIV Tat and ethanol for 24 h. Consistent with observations in MPAs, coexposure of HIV Tat (50 ng/mL) and ethanol (50 mM) significantly increased GFAP expression (Figure S2A) as well as NLRP6 inflammasome signaling components, including NLRP6 and cleaved caspase‐1 (p20) (Figure S2B,C). In addition, coexposure of HIV Tat and ethanol elevated the protein levels of proinflammatory cytokines IL‐1β and IL‐18, as assessed by western blotting (Figure S2D,E). Collectively, these findings demonstrate that ethanol enhances HIV Tat‐induced NLRP6 inflammasome activation and promotes the release of proinflammatory cytokines in astrocytes.

**FIGURE 1:**
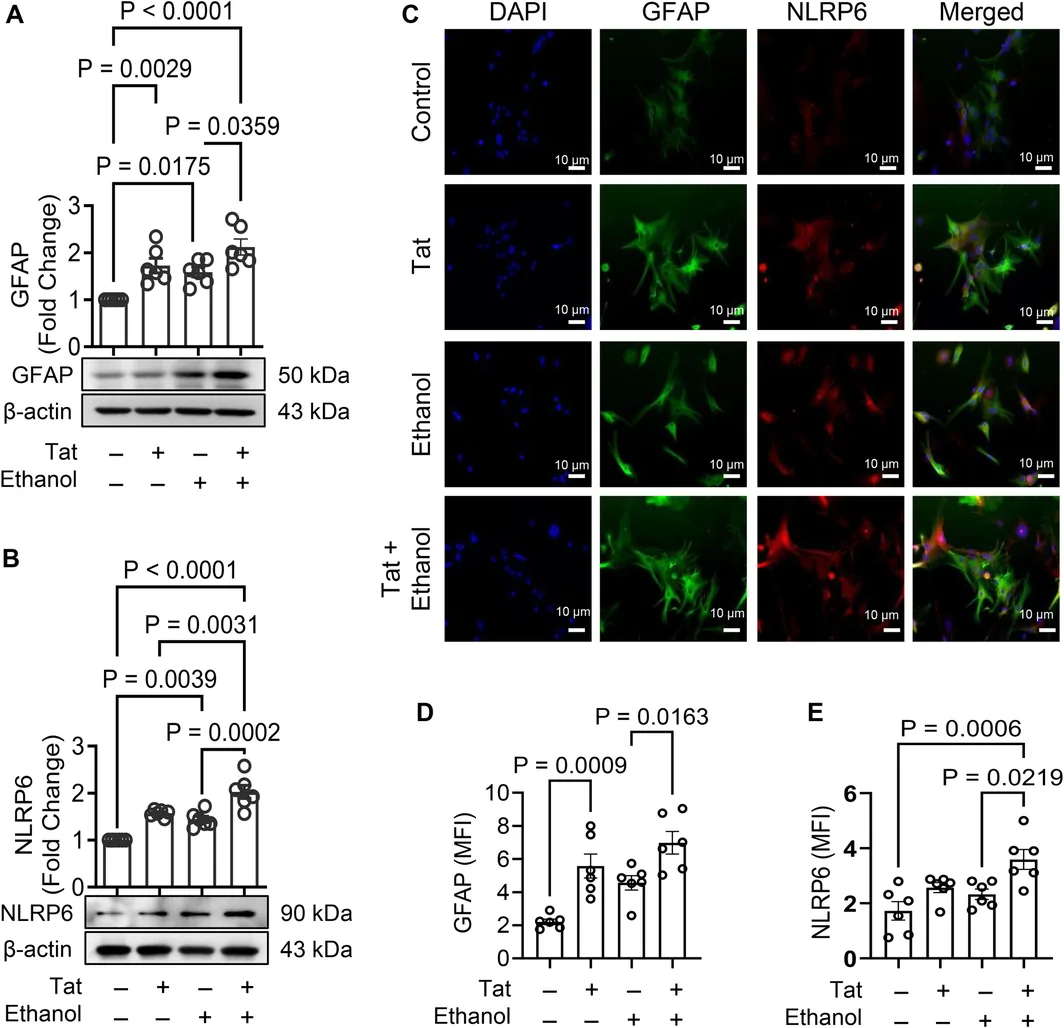
HIV Tat and ethanol together enhance NLRP6 and GFAP expression in MPAs. Representative western blots and corresponding graphs showing increased expression of GFAP (A) and NLRP6 (B) in MPAs exposed to HIV Tat (50 ng/mL) and ethanol (50 mM) in combination for 24 h. β‐actin was used as a loading control. (C–E) Immunofluorescence staining and corresponding bar graph showing activation of astrocytes (GFAP; green), NLRP6 (red), and DAPI (blue) in HIV Tat, and ethanol‐exposed MPAs. Scale bar: 10 μm. Data from six independent experiments are represented as mean ± SEM and analyzed using one‐way ANOVA following Sidak's multiple comparisons test.

**FIGURE 2:**
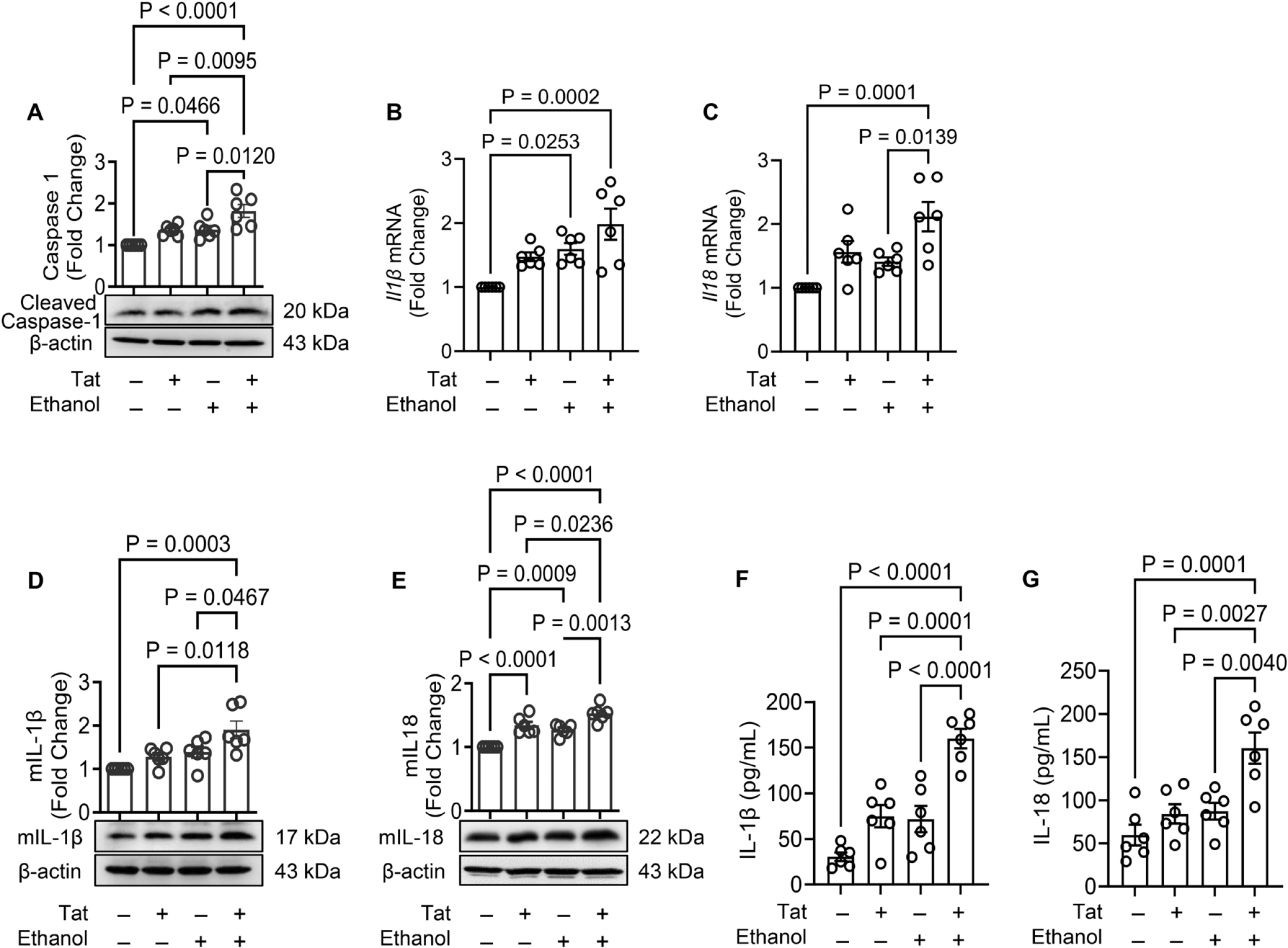
HIV Tat and ethanol together activate NLRP6 signaling mediators in MPAs. Representative western blots and corresponding graphs showing increased protein expression of cleaved caspase 1 (A) in MPAs exposed to HIV Tat (50 ng/mL) and ethanol (50 mM) for 24 h. Representative bar graph showing increased pro‐inflammatory cytokines such as *Il1β* and *Il18* mRNAs, as confirmed by qPCR (B and C), and their protein levels, as determined by western blotting (D and E) and ELISA (F and G), in MPAs exposed to HIV Tat (50 ng/mL) and ethanol (50 mM) for 24 h. β‐actin was used as a loading control for western blotting, and *gapdh* was used as a positive control for qPCR analysis. Data from six independent experiments are represented as mean ± SEM and analyzed using one‐way ANOVA following Sidak's multiple comparisons test.

### 3.2 HIV Tat and Ethanol Cooperatively Induce ER Stress in Astrocytes

Given that inflammasome activation is closely linked to intracellular stress responses, we next examined whether the combined exposure to HIV Tat and ethanol induces ER stress in astrocytes. Coexposure with HIV Tat and ethanol markedly increased the expression of BiP/GRP78, a canonical ER‐stress marker, compared to individual exposure in both MPAs and human A172 astrocytoma cell lines (Figures 3A and S2F). Moreover, western blotting analysis revealed significant upregulation of the downstream UPR effectors phosphorylated eIF2α (p‐eIF2α) and ATF6 following coexposure (Figure 3B,C and S2G). To further validate the role of ER stress in this process, MPAs were pretreated with the ER‐stress inhibitor 4‐phenylbutyric acid (4‐PBA; 25 μM, 1 h before HIV Tat and ethanol exposure). 4‐PBA pretreatment markedly attenuated the induction of BiP (Figure 3D), p‐eIF2α (Figure 3E), and ATF6 (Figure 3F), confirming that HIV Tat and ethanol cooperatively activate ER‐stress pathways in astrocytes.

**FIGURE 3:**
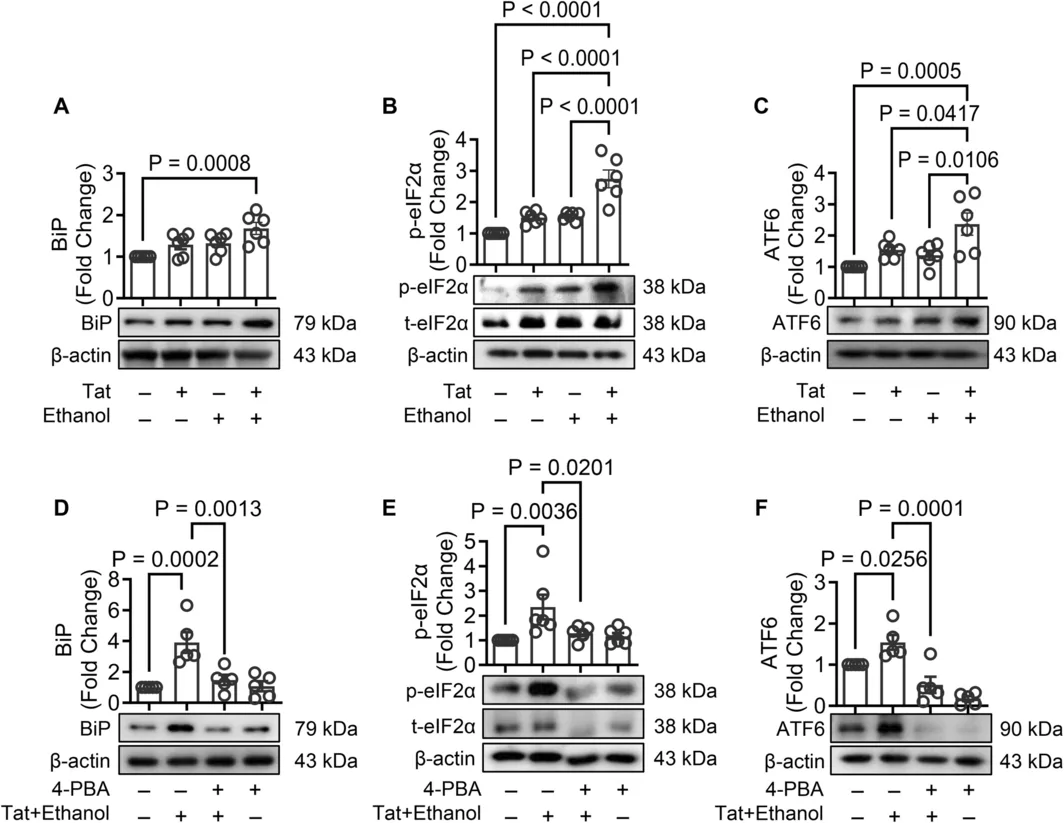
HIV Tat and ethanol together enhanced ER stress compared to their individual exposure in MPA cells. Representative western blots and corresponding graphs showing increased protein expression of the ER stress marker BiP (A) and the downstream effector molecules‐p‐eIF2α (B) and ATF6 (C) in the MPA exposed to HIV Tat (50 ng/mL) and ethanol (50 mM) together. Representative western blots and corresponding graphs showing reduced expression of the ER stress marker BiP (D), ER stress downstream pathways of p‐eIF2α (E), and ATF6 (F) in MPA cells pre‐treated with 4‐PBA (25 μM) 1 h before exposure to HIV Tat and ethanol. β‐actin was used as a loading control. Data from six independent experiments are represented as mean ± SEM and analyzed using one‐way ANOVA following Sidak's multiple comparisons test.

### 3.3 ER Stress Upregulates NLRP6 Inflammasome Activation in Response to HIV Tat and Ethanol in Astrocytes

To determine whether ER stress functions upstream of NLRP6 inflammasome activation, astrocytes were pretreated with 4‐PBA prior to HIV Tat and ethanol exposure. Pharmacological inhibition of ER stress significantly suppressed the upregulation of NLRP6 protein (Figure 4A) and reduced the activation of downstream signaling mediators, including cleaved caspase‐1 (Figure 4B), *Il1β* (Figure 4C), and *Il18* (Figure 4D) mRNAs, as well as mature IL‐1β (Figure 4E) and IL‐18 (Figure 4F) proteins. Additionally, 4‐PBA pretreatment diminished GFAP expression, indicating reduced astrocytic activation (Figure 4G). Immunostaining analysis further confirmed that HIV Tat and ethanol exposure increased the expression and colocalization of NLRP6 with GFAP, while 4‐PBA pretreatment significantly alleviated these effects, further supporting the involvement of upstream ER stress signaling in NLRP6 inflammasome activation (Figure 4H–J). To further verify the directionality of this signaling pathway, we examined ER‐stress markers in NLRP6‐silenced MPAs. Knockdown of NLRP6 using siRNA effectively reduced NLRP6 expression (Figure 5A) but did not alter the levels of BiP (Figure 5B), p‐eIF2α (Figure 5C), or ATF6 (Figure 5D) following HIV Tat and ethanol exposure. These results demonstrate that ER stress functions as an upstream regulator of NLRP6 inflammasome activation and that its inhibition attenuates HIV Tat‐ and ethanol‐induced inflammatory responses.

**FIGURE 4:**
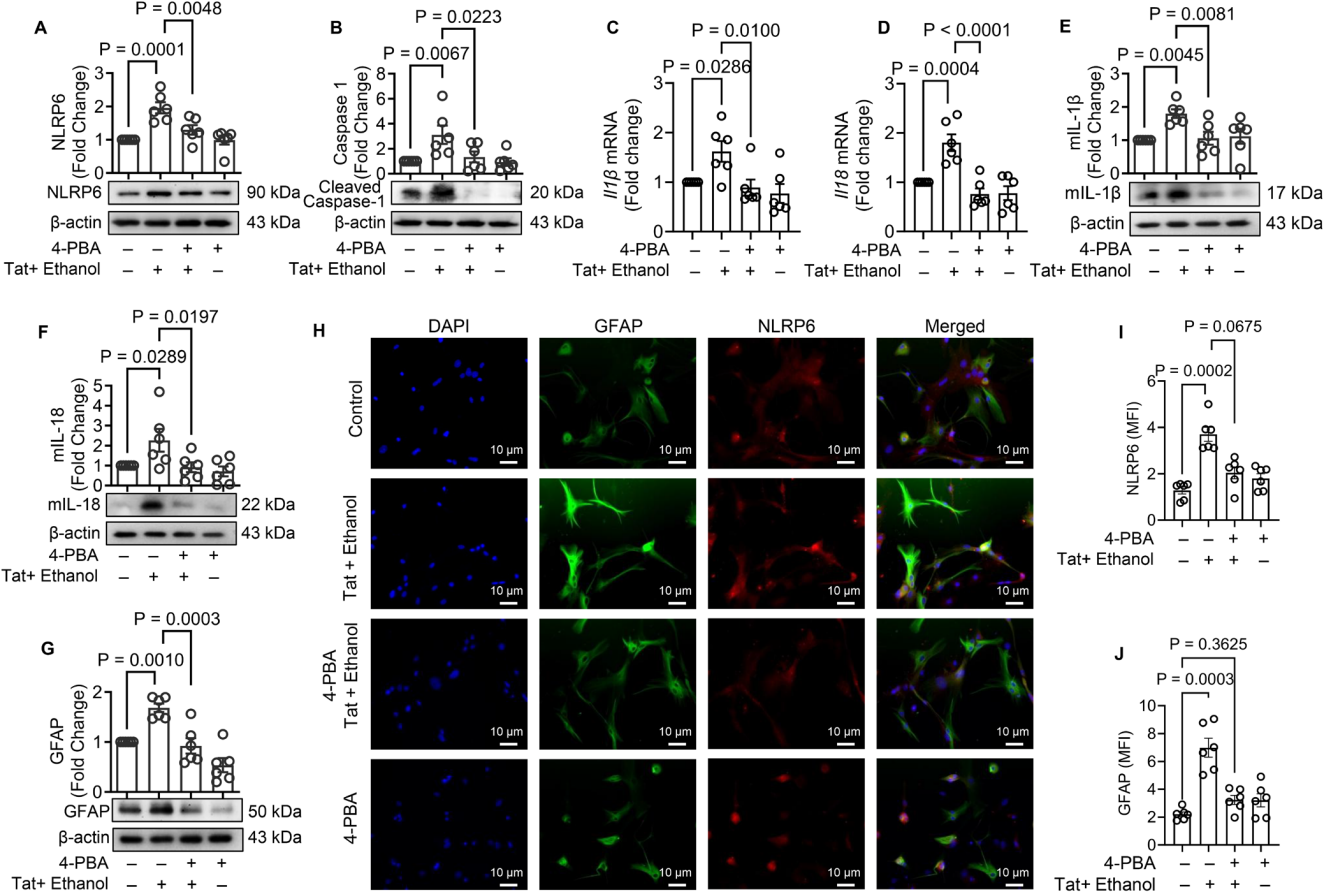
ER stress is upstream to HIV Tat and ethanol‐mediated activation of NLRP6 signaling in MPA cells. Representative western blots and corresponding graphs showing decreased expression of NLRP6 (A) and downstream mediator cleaved caspase‐1 (B) in MPAs pre‐treated with 4‐PBA (25 μM) 1 h before exposure to HIV Tat (50 ng/mL) and ethanol (50 mM) for 24 h. Representative bar graph showing decreased expression of pro‐inflammatory cytokines such as cytokines *Il1β* and *Il18*, both at mRNA (C and D), and protein levels (E and F) in MPAs exposed to HIV Tat (50 ng/mL) and ethanol (50 mM) for 24 h. Representative blot and corresponding quantification showing decreased expression of astrocyte activation marker GFAP (G) in MPAs exposed to HIV Tat (50 ng/mL) and ethanol (50 mM) for 24 h. Representative immunofluorescence staining and corresponding bar graph showing expression of NLRP6 (red), GFAP (green), and DAPI (blue) in MPAs pre‐treated with 4‐PBA (25 μM) 1 h before exposure to HIV Tat (50 ng/mL) and ethanol (50 mM) for 24 h (H–J). Scale bar: 10 μm. β‐Actin was used as a loading control for western blotting, and *gapdh* was used as a positive control for qPCR analysis. Data from six independent experiments are represented as mean ± SEM and analyzed using one‐way ANOVA following Sidak's multiple comparisons test.

**FIGURE 5:**
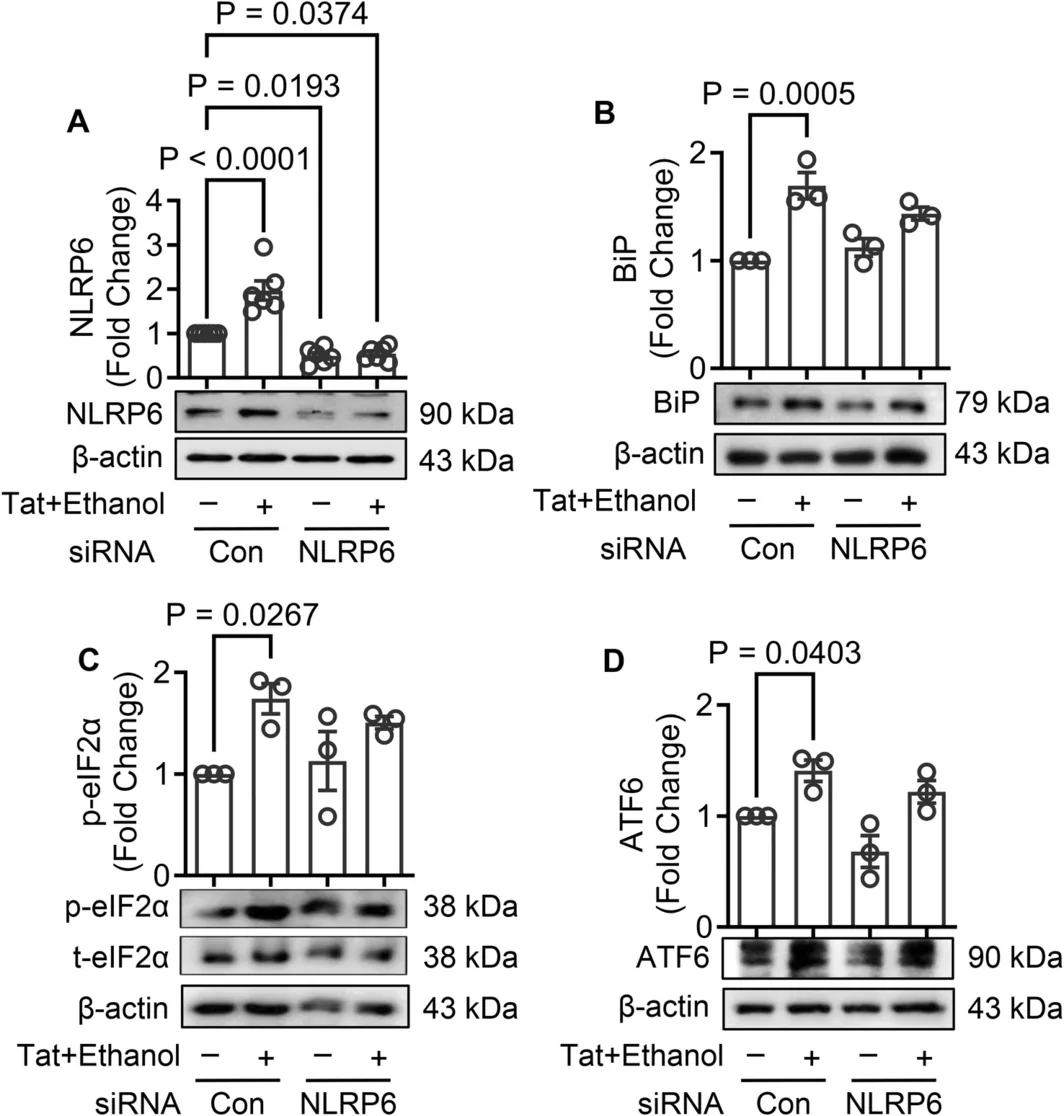
Silencing NLRP6 did not affect HIV Tat and ethanol‐induced ER stress in MPA cells. (A) Representative western blots and corresponding graphs showing transfection efficiency of NLRP6 in scrambled or NLRP6 siRNA‐transfected MPAs exposed to HIV Tat (50 ng/mL) and ethanol (50 mM) for 24 h. Representative western blots and corresponding graphs showing no change in expression of ER stress markers‐BiP (B), p‐eIF2α (C), and ATF6 (D) between scrambled or NLRP6 siRNA‐transfected MPAs exposed to HIV Tat (50 ng/mL) and ethanol (50 mM) together. β‐actin was used as a loading control for western blotting. Data from six independent experiments are represented as mean ± SEM and analyzed using one‐way ANOVA following Sidak's multiple comparisons test.

### 3.4 NLRP6 Knockdown Attenuates HIV Tat‐ and Ethanol‐Mediated Inflammasome Activation and Cytokine Release

To confirm the causal role of NLRP6 in mediating astrocytic inflammation, siRNA‐mediated silencing was employed. NLRP6 knockdown also significantly decreased the expression of cleaved caspase‐1 (Figure 6A), as well as the mRNA and protein levels of IL‐1β and IL‐18 (Figure 6B–E). Furthermore, NLRP6 silencing markedly reduced GFAP expression (Figure 6F), indicating diminished astrocytic activation. Together, these results establish NLRP6 as a critical mediator of HIV Tat‐ and ethanol‐induced inflammasome activation and proinflammatory cytokine release in astrocytes. Overall, our results reveal that HIV Tat and ethanol synergistically activate ER‐stress and NLRP6‐inflammasome signaling in astrocytes, leading to caspase‐1 cleavage and proinflammatory cytokine release. Pharmacological inhibition of ER stress with 4‐PBA suppresses this signaling cascade, while genetic knockdown of NLRP6 reduces inflammasome activation and astrocyte reactivity. These findings identify ER stress as an upstream regulator of NLRP6‐mediated neuroinflammation in astrocytes under HIV Tat and alcohol coexposure.

**FIGURE 6:**
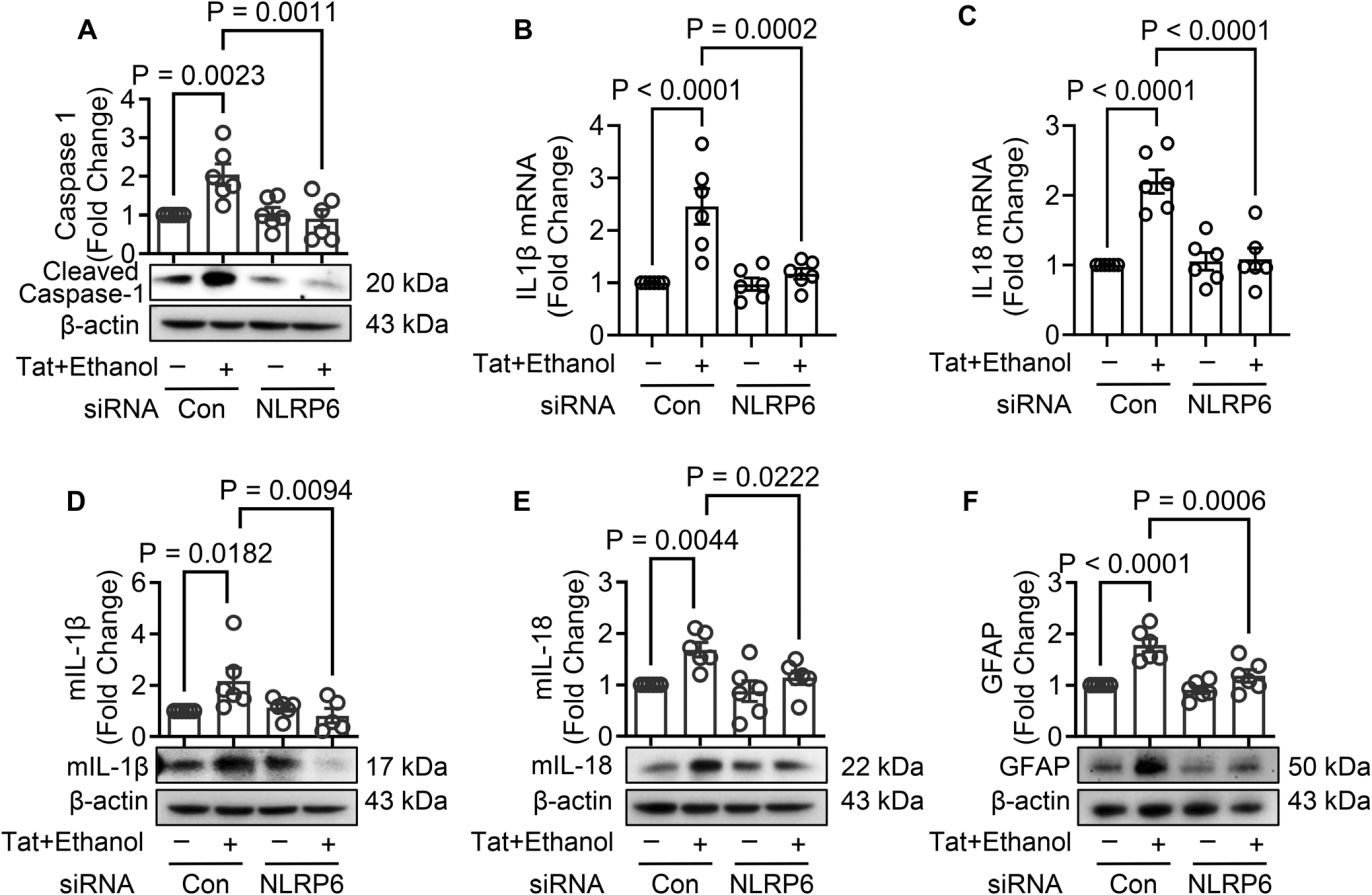
Silencing NLRP6 signaling inhibited HIV Tat and ethanol‐mediated activation of MPAs. (A) Representative western blots and corresponding graphs demonstrating significant inhibition of NLRP6 inflammasome signaling mediators such as cleaved caspase 1 in NLRP6‐silenced MPAs exposed to HIV Tat (50 ng/mL) and ethanol (50 mM) for 24 h. (B–E) Representative graphs demonstrating silencing of NLRP6 in MPAs attenuated HIV Tat (50 ng/mL) and ethanol (50 mM; 24 h) mediated upregulation of proinflammatory cytokines, *Il1β* and *Il18* mRNA transcript (B and C) and their protein level (D and E). (F) Representative western blot and corresponding graph demonstrating downregulation of GFAP expression in NLRP6‐silenced MPAs exposed to HIV Tat (50 ng/mL) and ethanol (50 mM) for 24 h. β‐actin was used as a loading control for western blotting, and *gapdh* was used as a positive control for qPCR analysis. Data from six independent experiments are represented as mean ± SEM and analyzed using one‐way ANOVA following Sidak's multiple comparisons test.

## 4 Discussion

The present study identifies a novel signaling axis in which HIV Tat and ethanol cooperatively induce ER stress and activate the NLRP6 inflammasome in astrocytes, leading to proinflammatory cytokine release and astrocytic reactivity. This ER stress‐NLRP6 inflammasome signaling pathway represents a previously uncharacterized mechanism contributing to neuroinflammation in the context of NeuroHIV and AUD. By demonstrating that pharmacological inhibition of ER stress suppresses inflammasome activation and proinflammatory cytokine production, while NLRP6 knockdown attenuates downstream inflammatory responses, our findings establish ER stress as an upstream regulator of astrocytic inflammasome signaling in HIV and alcohol coexposure conditions.

ER stress is a key cellular adaptive mechanism triggered by perturbations in protein folding, calcium homeostasis, and redox balance [44, 45, 46, 47]. When sustained, it initiates the UPR, which engages signaling pathways mediated by PERK, IRE1α, and ATF6 to restore proteostasis or, when unresolved, to promote inflammation and cell death [48]. Both HIV Tat and ethanol are individually known to disrupt ER homeostasis through oxidative and metabolic stress [49, 50]. In fact, emerging evidence in AUD indicates that ethanol exposure is associated with ER stress in astrocytes and even influences drug‐craving networks in astrocytic models via IRF3, STING, and GRP78 signaling [51]. For example, a recent genomic study of iPSC‐derived astrocytes from AUD patients revealed that ethanol‐induced ER stress was linked to IRF3 activation and could be attenuated by anticraving agents [51]. Consistent with these reports, our results show that HIV Tat (50 ng/mL) and ethanol (50 mM) coexposure synergistically elevate canonical ER stress markers BiP/GRP78, p‐eIF2α, and ATF6. The concentrations used in this study are consistent with established in vitro models of HIV‐associated neuroinflammation [52, 53, 54, 55] and alcohol exposure [12]. HIV Tat in the range of 10–100 ng/mL has been shown to induce inflammatory and neurotoxic responses in glial cells [40, 41, 42, 43, 56]. Moreover, extracellular Tat has been detected in the CSF of cART‐treated individuals at ~5–35 ng/mL [57], with brain tissue levels likely exceeding those measured in CSF. Similarly, ethanol concentrations of 25–100 mM are widely used to model moderate to heavy alcohol exposure and associated neuroinflammatory responses in brain cells [58]. Furthermore, this convergence of viral and toxicological stressors could likely overwhelm astrocytic proteostasis, amplifying inflammatory responses and thus contributing to neuropathological outcomes in PLWH with concurrent alcohol use.

While inflammasome activation in the CNS has been widely attributed to the NLRP3 complex [20, 21, 22, 59, 60], our study underscores the emerging role of NLRP6 in astrocytic innate immune signaling. NLRP6, originally characterized in intestinal epithelial cells as a regulator of microbial homeostasis and sterile inflammation, has recently been recognized in glial populations under neurodegenerative and substance‐induced stress conditions [61, 62]. Our earlier study also showed that ethanol exposure upregulated NLRP6 signaling mediators in human primary astrocytes, including caspase‐1, IL‐1β, and IL‐18, and identified miR‐339 as a negative regulator of NLRP6 in that context [12]. Our data demonstrate that NLRP6 is upregulated in astrocytes following HIV Tat and ethanol exposure, paralleling increases in caspase‐1 activation and IL‐1β and IL‐18 release. Importantly, both pharmacological suppression of ER stress and siRNA‐mediated NLRP6 silencing abrogated these responses, defining a mechanistic hierarchy wherein ER stress precedes inflammasome activation. The persistence of ER stress markers despite NLRP6 knockdown further confirms that ER perturbation acts upstream of inflammasome signaling in this paradigm. Another recent study on methamphetamine exposure also demonstrated a central role of NLRP6‐mediated astrocytic pyroptosis and proinflammatory responses, highlighting the broader relevance of NLRP6 across substance‐induced neuroinflammation [18].

These findings align with prior studies demonstrating that chronic ER stress can act as a trigger for inflammasome activation through mechanisms that include mitochondrial dysfunction, ROS accumulation, calcium dysregulation, and hemichannel‐mediated ATP release [63, 64]. For instance, an investigation of ethanol‐exposed astrocytes uncovered that Cx43 and Panx1 hemichannels contributed to mitochondrial superoxide production and astrocyte dysfunction, linking to inflammatory pathways [63, 65]. In astrocytes, the UPR may promote inflammatory gene transcription via ATF6 and CHOP, thereby priming inflammasome components and facilitating cytokine maturation [35]. Our results suggest that in the context of HIV Tat and ethanol exposure, excessive ER stress serves as a molecular bridge between cellular proteostasis failure and innate immune activation. This mechanism expands current understanding of astrocyte‐mediated neuroinflammation beyond the canonical NLRP3 axis and introduces NLRP6 as a novel player in glial pathology associated with NeuroHIV and AUD.

The inflammatory cytokines IL‐1β and IL‐18, downstream effectors of inflammasome activation, are critical mediators of neuroimmune signaling. Elevated IL‐1β levels have also been implicated in neuronal injury, synaptic dysfunction, and blood–brain barrier disruption [66, 67, 68, 69, 70]. In the present study, HIV Tat and ethanol coexposure significantly increased both the proinflammatory cytokines at the transcriptional and protein levels, reinforcing the proinflammatory state of astrocytes. Considering the emerging literature on astrocyte‐microglia crosstalk, such as an elegant review on glial neuroinflammation which emphasizes the bidirectional signaling between astrocytes and microglia in disease contexts, our data may reflect an astrocyte‐initiated inflammatory cascade that could amplify microglial responses [71]. Given the established role of IL‐1β and IL‐18 in promoting neuronal toxicity and cognitive impairment, persistent activation of this ER stress‐NLRP6 inflammasome signaling axis may underlie the neuropathological progression observed in individuals with comorbid HIV infection and alcohol abuse.

Beyond astrocytes, NLRP6 has broader relevance to systemic inflammation and gut‐brain axis regulation [23, 72]. Previous studies have shown that NLRP6 deficiency alters gut microbial composition and heightens susceptibility to inflammatory insults [72]. Considering the bidirectional communication between the gut and CNS, and the known effects of both HIV and alcohol on gut permeability and microbial translocation, NLRP6 may serve as a molecular conduit linking peripheral and central immune responses. Given recent findings that alcohol exposure engages purinergic P2X7 receptor and extracellular vesicle signaling to compromise the blood–brain barrier and trigger neuroinflammation, the role of inflammasomes like NLRP6 in connecting gut‐brain and glial pathways warrants future investigation [73].

From a mechanistic perspective, our findings identify ER stress as an upstream regulator of NLRP6 inflammasome activation in astrocytes. While pharmacological attenuation of ER stress using 4‐PBA supports this relationship, it does not distinguish the relative contribution of individual UPR branches (PERK, IRE1, or ATF6). Thus, although ER stress modulation may represent a potential strategy to mitigate astrocyte‐driven inflammation, further studies using pathway‐specific approaches will be required to define the precise signaling mechanisms involved. Targeted modulation of NLRP6 may also provide a complementary approach to regulate inflammasome‐driven neuroinflammatory responses. Several other limitations should also be acknowledged as this study was conducted using MPAs and human A172 astrocytoma cell line, which may not fully recapitulate the complexity of human primary astrocyte responses. NLRP6 is evolutionarily conserved across multiple species, including mouse, rat, nonhuman primates, and humans, supporting the broader biological relevance of this pathway. Future studies using human iPSC‐derived astrocytes or postmortem brain tissues, along with in vivo models, will be necessary to establish physiological relevance.

In summary, our study demonstrates that HIV Tat and ethanol synergistically induce ER stress‐dependent NLRP6 inflammasome activation in astrocytes, leading to increased IL‐1β and IL‐18 production and promoting neuroinflammatory responses. These findings define an ER stress‐NLRP6 signaling axis as a potential mechanism linking HIV Tat and ethanol exposure to astrocyte dysfunction, providing mechanistic insight into NeuroHIV in the context of substance use.

## Author Contributions

**Seema Singh:** writing – review and editing, writing – original draft, visualization, validation, methodology, investigation, funding acquisition, formal analysis, data curation. **Elias Horanieh:** writing – review and editing, validation, methodology. **Shilpa Buch:** writing – review and editing, supervision, resources. **Palsamy Periyasamy:** writing – review and editing, visualization, supervision, resources, funding acquisition, conceptualization.

## Funding

This study was supported by the National Institute on Alcohol Abuse and Alcoholism (AA031444; P50AA030407‐5126, Pilot Core grant) to S.S. and startup funding from the University of Nebraska Medical Center to P.P.

## Ethics Statement

The animal study was approved by The Institutional Animal Care and Use Committee (IACUC) of the University of Nebraska Medical Center (Approval #18‐030‐04‐FC). The study was conducted in accordance with the local legislation and institutional requirements.

## Conflicts of Interest

The authors declare no conflicts of interest.

## Supporting information


**Figure S1:** Purity of cultured mouse primary astrocytes. Representative immunofluorescence images of mouse primary astrocytes isolated from 1 to 3‐days‐old pups and cultured for 10 days in vitro. Cells were stained for GFAP (green, astrocytes), IBA‐1 (red, microglia), MAP2 (pink, neurons), and DAPI (blue, nuclei) to assess cellular purity. Scale bar: 20 μm.
**Figure S2:** HIV‐1 Tat and ethanol coexposure activates the ER stress‐NLRP6 axis in human A172 astrocytoma cell lines. (A–G) Representative Western blot images and corresponding densitometric quantification showing the expression of GFAP (A), NLRP6 (B), and cleaved caspase‐1 (C), pro‐inflammatory cytokines IL‐1β (D) and IL‐18 (E); and ER stress markers BiP (F) and p‐eIF2α (G) in human A172 astrocytoma cell lines following exposure to HIV Tat (50 ng/mL) and ethanol (50 mM) for 24 h.

## Data Availability

The data that support the findings of this study are available from the corresponding author upon reasonable request.
